# The association of lymphocyte with hypothyroidism in obstructive sleep apnea

**DOI:** 10.1186/s12890-024-02872-7

**Published:** 2024-01-27

**Authors:** Xiaoyan Fang, Le Wang, Chong Xu, Tuai Xue, Mingchu Zhang, Lingling Liu, Jie Cao, Jing Zhang

**Affiliations:** Department of Respiratory and Critical Care Medicine, Tianjin Medical University General Hospital, Tianjin, China

**Keywords:** Obstructive sleep apnea, Hypothyroidism, Lymphocyte, Hashimoto's thyroiditis

## Abstract

**Purpose:**

Obstructive sleep apnea (OSA) is a common sleep-breathing disorder. Numerous investigations have found a strong inherent relationship between OSA and hypothyroidism. Studies suggest that lymphocytes may be involved in the development of hypothyroidism in patients with OSA. This study aimed to assess the association between lymphocytes and hypothyroidism in OSA patients.

**Patients and methods:**

This study involved 920 patients with OSA who underwent nocturnal sleep monitoring, thyroid function testing, and routine blood tests. In patients with OSA, logistic regression analysis indicated independent predictors of hypothyroidism. The cutoff level of lymphocyte count was determined using a receiver operating characteristic (ROC) analysis to predict the occurrence of hypothyroidism in individuals with OSA.

**Results:**

This study comprised 920 OSA patients (617 males and 303 women), 879 with normal thyroid function, and 41 with hypothyroidism, with a hypothyroidism incidence of 4.46%. In the entire OSA population and male OSA patients, the number of lymphocytes was significantly higher in the hypothyroid group than in the control group (*p* = 0.002 and 0.020, respectively). In addition, among the OSA population younger than 60 years old and patients with mild to moderate OSA, lymphocytes were found to be considerably more in the hypothyroid group than in the euthyroid group. Lymphocyte count, ESS, and sex were all independent predictors of hypothyroidism development in OSA patients. According to ROC curve analysis, the risk of hypothyroidism increases with increasing lymphocyte count in the total patient population, with an optimal diagnostic cutoff point of 2.5 (× 10*9/L).

**Conclusions:**

The prevalence of hypothyroidism in patients with OSA increases as the number of lymphocytes increases. Lymphocyte count can be used as an independent predictor of the occurrence of hypothyroidism, and it has a diagnostic value for OSA combined with hypothyroidism.

## Introduction

Obstructive sleep apnea is a condition in which the periodic collapse of the upper airway during sleep causes complete or partial airway obstruction, resulting in apnea and hypoventilation [1]. Studies have shown that the prevalence of OSA in the adult population is approximately 9%-38%, fluctuates between 13%-33% in men, and is approximately 6%-19% in the female population [2]. Nocturnal intermittent hypoxia can lead to exacerbation of neurologic, cardiovascular, endocrine-metabolic, and other systemic disorders, increasing the risk of death. [3] All-cause mortality in untreated patients with severe OSA (AHI > 30) was 3.8 times higher than in those without sleep apnea [4]. Studies have shown that common comorbidities of OSA are hypertension, arrhythmia, coronary artery disease, and diabetes [5]. This shows that OSA can cause a significant health hazard and disease burden.

Hypothyroidism is a common disorder of the endocrine system. In iodine-sufficient countries, the prevalence of clinical hypothyroidism is approximately 1% to 2% [6]. The prevalence of subclinical hypothyroidism has been reported to be 4%-20% in the adult population [7]. Hypothyroidism is most often caused by autoimmunity. In addition, thyroid surgery, radioactive iodine treatment, and related medications are essential causes of hypothyroidism [7, 8]. Chronic lymphocytic thyroiditis is an autoimmune disease of the thyroid gland and is the most common cause of hypothyroidism [8]. Hashimoto's thyroiditis (HT) is a familiar type of autoimmune thyroiditis characterized by T lymphocyte infiltration of target tissues. It mediates the process of target cell destruction and apoptosis, resulting in hypofunction of the target gland [9]. Th1 and Th17 lymphocytes have been shown to infiltrate thyroid tissue and mediate thyroid cell death in autoimmune thyroiditis [10].

Many previous studies have confirmed the close association between lymphocytes and hypothyroidism [9, 11]. Lymphocytes are involved in the process of hypothyroidism, especially in autoimmune diseases of the thyroid, such as Hashimoto's thyroiditis. However, the relationship between lymphocytes and hypothyroidism in patients with OSA is unclear. This study aimed to assess the association of lymphocytes with hypothyroidism in patients with OSA, which has not been studied previously.

## Data and methods

### Study population

**Fig. 1:**
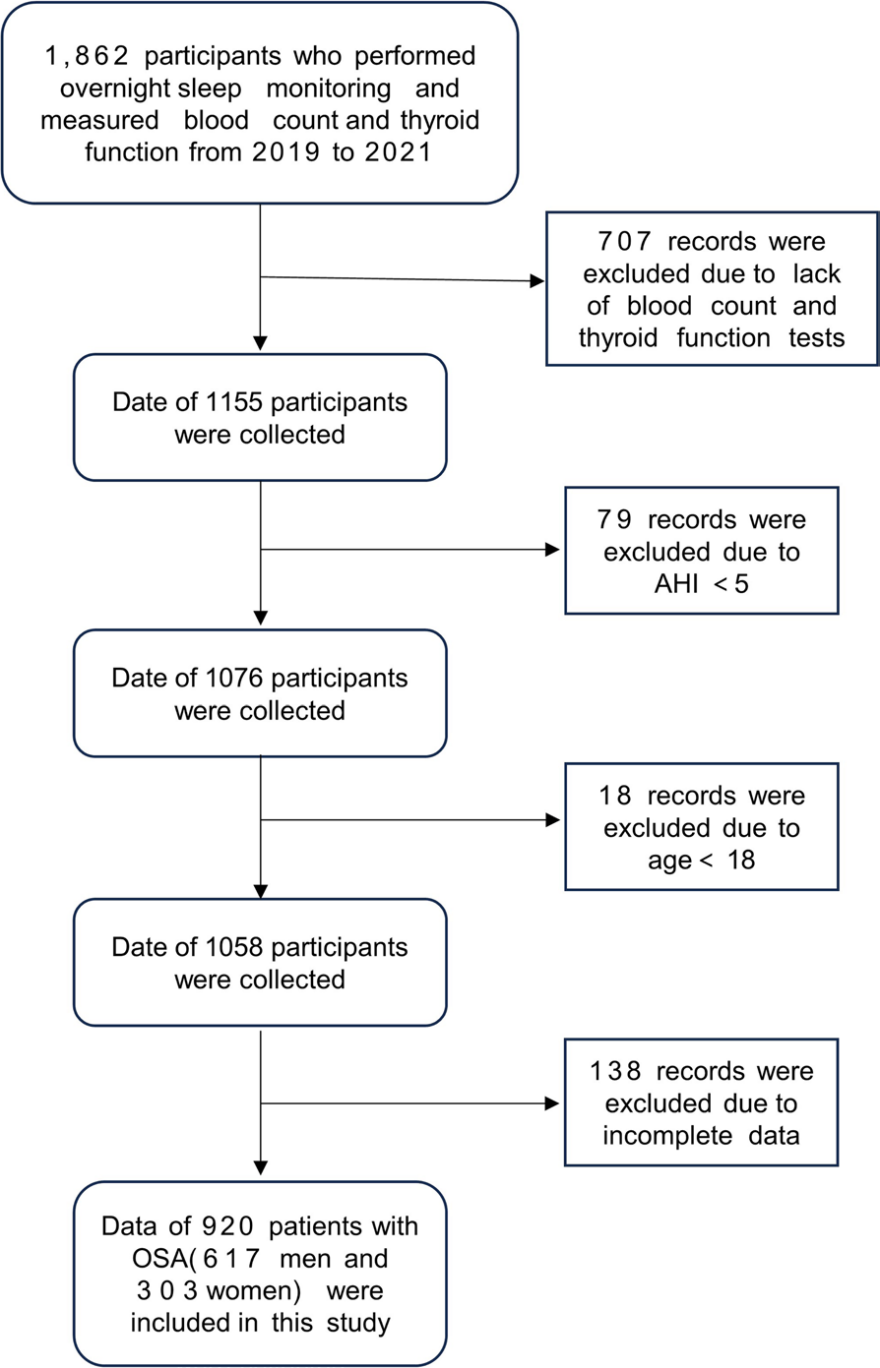
Flow chart of study population

This is a retrospective study. The participants in this study were all from the Sleep Center of the Tianjin Medical University General Hospital. Nine hundred twenty patients with OSA were enrolled in the study (Fig. 1). All patients underwent standard polysomnography using an analysis system (Alice 5 Diagnostic Sleep System; Philips Respironics, Bend, OR, USA) in the sleep center.

### Inclusion and exclusion criteria

The inclusion criteria were as follows: 1) complete overnight sleep monitoring who met the diagnostic criteria for obstructive sleep apnea; 2) completed blood cell analysis and free thyroid function tests;and 3) complete baseline information, such as height, weight, neck circumference, and waist circumference.

The exclusion criteria were as follows: 1) less than 18 years old; 2) incomplete information on clinical data; 3) patients with previously defined thyroid function abnormalities who were taking thyroid-related therapeutic drugs; 4) patients with newly diagnosed hyperthyroidism; 5) patients with central sleep apnea; 6) patients with hematologic disorders that may affect lymphocyte counts; and 7) patients with a recent history of definite infection-related illness.

### Data information

All clinical data of polysomnography, thyroid function, and routine blood tests of OSA patients in this study were obtained from the Sleep Center of Tianjin Medical University General Hospital.

### Diagnostic criteria

Patients with an apnea hypopnea index (AHI) score ≥ 5 were diagnosed with OSA. Patients with OSA were divided into three groups according to AHI scores: mild, moderate, and severe OSA (5–15, > 15–30, and > 30, respectively) [12]. Assessment of thyroid function in OSA patients by free thyroid function measurement. Normal thyroid function means FT3, FT4, and TSH levels are within normal limits. In this study, hypothyroidism included overt hypothyroidism and subclinical hypothyroidism. Overt hypothyroidism is defined as reduced FT4 and increased TSH levels. Subclinical hypothyroidism is elevated TSH levels with FT4 levels in the normal range [13]. In the present study, patients with hyperthyroidism were excluded. Hyperthyroidism includes overt hyperthyroidism and subclinical hyperthyroidism. The standard reference ranges of free thyroid function parameters were as follows: FT3 2.43–6.01 pmol/L, FT4 9.01–19.05 pmol/L, and TSH 0.350–4.940 uIU/mL. The Epworth sleepiness scale is a daytime sleepiness assessment tool with a total score of 24 [14]. An ESS score ≥ 9 suggests the presence of daytime sleepiness.

### Statistical analysis

Data were analyzed using the Windows SPSS 25.0 statistical software package (IBM SPSS, Armonk, NY, USA). Normally distributed data are expressed as the mean ± standard deviation, and nonnormally distributed data are expressed using the median and quartiles. The Shapiro–Wilk test was used to test whether the data obeyed a normal distribution. If the data were normally distributed, the Student's test was used, and if the data did not conform to a normal distribution, the Mann–Whitney U test was used. Binary logistic regression was performed to determine the predictors of hypothyroidism in patients with OSA. Receiver operating characteristic (ROC) curve analysis was performed to determine the cutoff lymphocyte count to predict the occurrence of hypothyroidism in OSA patients. The chi-square test was used to compare differences in prevalence between groups. A value of *p* < 0.05 was considered statistically significant. GraphPad Prism 9.5 (GraphPad, San Diego, CA, USA) was used to generate figures.

## Results

### Baseline information and clinical data of the study population

#### Total population data characteristics

**Fig. 2:**
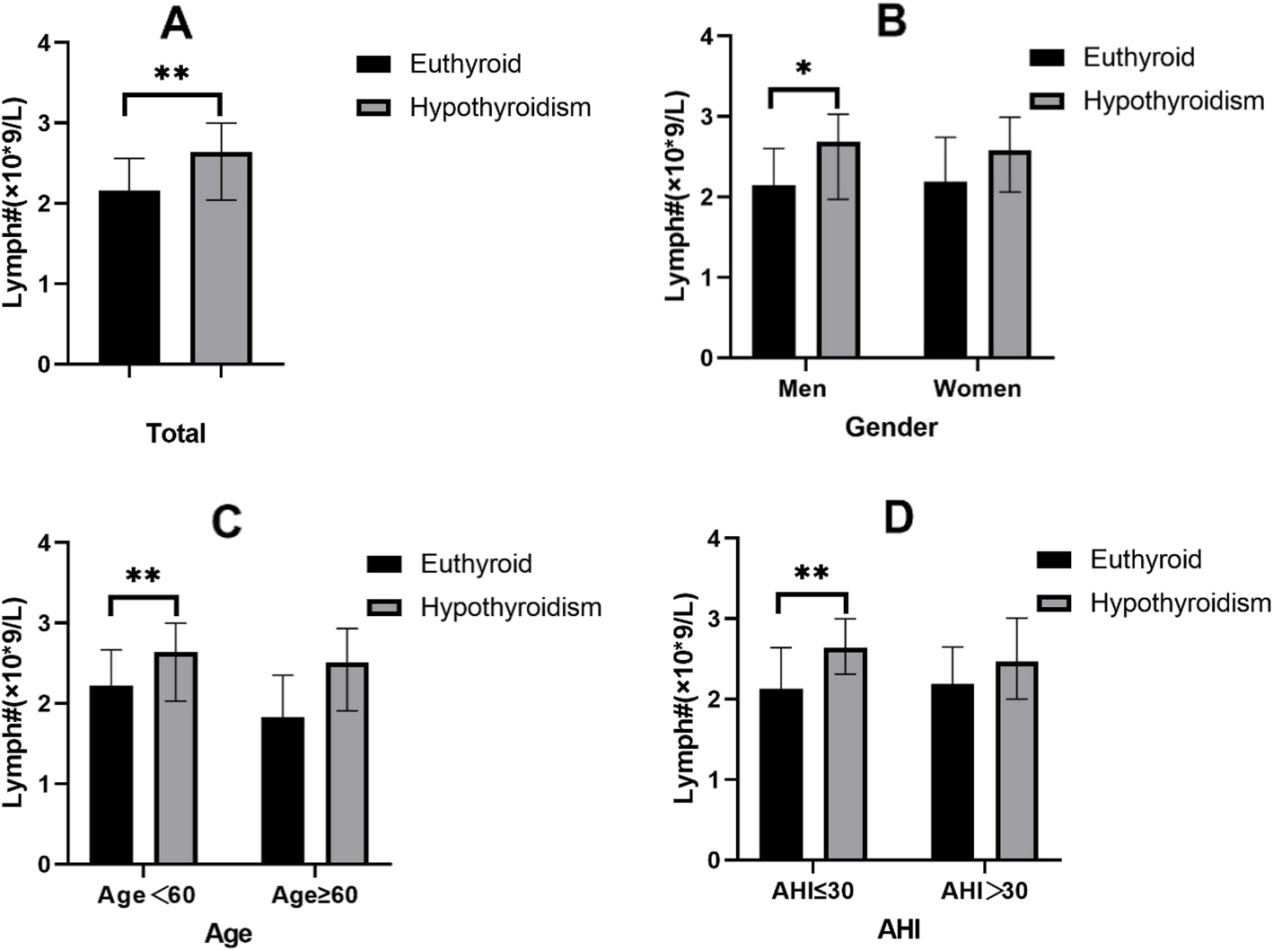
Differences in lymphocyte counts between the euthyroid and hypothyroid groups in different OSA population subgroups (*p < 0.05,**p < 0.01,***p < 0.001)

Baseline characteristics and clinical data are presented in Table 1. A total of 920 patients with OSA were enrolled in the study: 879 (mean age: 43.74 ± 14.06 years) with normal thyroid function and 41 (mean age: 41.63 ± 13.94 years) with newly diagnosed hypothyroidism. The BMIs of OSA patients with hypothyroidism and controls were 34.78 ± 8.99 and 32.38 ± 7.79, respectively. ESS, lymphocyte percentage, and lymphocyte count (Fig. 2A) were significantly higher in OSA patients with hypothyroidism than in controls.

**Table 1:** Baseline data characteristics of total, male and female OSA patients

	Total (*n* = 920)			Men (*n* = 617)			Women (*n* = 303)		
	Euthyroid	Hypothyroidism	P	Euthyroid	Hypothyroidism	P	Euthyroid	Hypothyroidism	P
Participant	879.00(95.54%)	41.00(4.46%)	-	596.00(96.60%)	21.00(3.40%)	-	283(93.40%)	20.00(6.60%)	-
Age	43.74 ± 14.06	41.63 ± 13.94	0.348	44.02 ± 13.13	42.05 ± 14.03	0.501	43.17 ± 15.84	41.20 ± 14.19	0.590
BMI	32.38 ± 7.79	34.78 ± 8.99	0.055	31.07 ± 7.11	31.67 ± 7.06	0.704	35.13 ± 8.42	38.06 ± 9.77	0.138
NC	41.87 ± 5.11	41.59 ± 4.07	0.725	42.89 ± 3.65	42.71 ± 3.15	0.829	39.72 ± 6.80	40.40 ± 4.65	0.662
WC	108.13 ± 16.36	111.16 ± 17.64	0.249	107.95 ± 16.06	109.29 ± 15.85	0.707	108.53 ± 16.99	113.13 ± 19.55	0.248
ESS	6.00(4.00, 12.00)	10.00(6.00, 14.50)	0.011	8.00(4.00, 14.00)	12.00(9.00, 15.00)	0.030	5.00(2.00, 9.00)	9.00(4.00, 12.50)	0.029
AHI	45.40(21.40, 71.70)	39.60(15.20, 77.45)	0.809	55.20(28.63, 73.45)	62.00(33.95, 80.40)	0.540	27.10(15.30, 48.90)	28.55(13.08, 53.38)	0.925
ODI	37.70(16.70, 66.10)	36.00(15.70, 66.90)	0.890	47.90(22.15, 70.10)	62.90(32.45, 77.75)	0.313	22.80(11.10, 45.60)	23.85(14.33, 35.93)	0.682
ArI	23.40(13.20, 43.90)	23.90(12.80, 45.30)	0.884	30.60(16.83, 47.60)	34.30(19.60, 52.70)	0.436	16.10(10.00, 25.20)	13.60(9.10, 27.88)	0.845
meanSpO2	94.00(92.00, 95.00)	94.00(90.00, 95.00)	0.391	94.00(91.00, 95.00)	92.00(89.50, 94.50)	0.084	95.00(93.00, 96.00)	95.00(92.25, 96.00)	0.847
miniSpO2	78.00(66.00, 85.00)	73.00(60.50, 85.50)	0.130	75.00(62.00, 83.75)	67.00(56.00, 79.50)	0.171	82.00(74.00, 87.00)	78.00(67.00, 86.75)	0.122
T90	4.90(0.30, 23.20)	7.25(0.40, 30.35)	0.583	8.90(1.00, 30.90)	24.80(3.25, 37.44)	0.192	0.90(0.02, 9.30)	1.25(0.12, 16.58)	0.634
FT3	4.50(4.14, 4.89)	4.43(4.06, 4.82)	0.360	4.58(4.22, 4.98)	4.55(4.04, 5.14)	0.976	4.30(3.98, 4.70)	4.23(4.053, 4.633)	0.581
FT4	12.39(11.51, 13.38)	11.91(10.88, 13.40)	0.071	12.40(11.55, 13.38)	12.05(11.06, 13.63)	0.690	12.34(11.44, 13.39)	11.36(10.85, 12.62)	0.032
TSH	1.88(1.31, 2.56)	5.88(5.25, 7.50)	< 0.001	1.74(1.20, 2.41)	5.88(5.21, 7.59)	< 0.001	2.11(1.52, 2.90)	5.84(5.28, 7.56)	< 0.001
WBC#(× 10^*^9/L)	6.76(5.61, 8.09)	7.48(5.97, 8.44)	0.146	6.68(5.65, 7.89)	7.51(5.99, 8.43)	0.202	7.01(5.55, 8.60)	7.30(5.66, 9.12)	0.568
RBC#(× 10^*^12/L)	4.87(4.52, 5.23)	4.85(4.57, 5.21)	0.634	5.06(4.72, 5.34)	5.04(4.83, 5.33)	0.603	4.56(4.26, 4.78)	4.63(4.20, 4.85)	0.743
HGB(g/L)	146.00(134.00, 156.00)	143.00(127.00, 155.00)	0.129	152.00(144.00, 159.00)	155.00(146.00, 159.50)	0.710	132.00(124.00, 138.00)	128.50(119.00, 133.50)	0.197
PLT#(× 10^*^9/L)	245.00(211.00, 288.00)	253.00(213.50, 296.00)	0.330	234.00(205.00, 270.00)	247.00(218.50, 282.00)	0.306	272.00(226.00, 323.00)	268.00(212.75, 316.50)	0.743
NEU%	56.10(50.70, 62.30)	54.50(49.25, 60.10)	0.165	55.75(50.40, 61.28)	52.90(48.65, 57.10)	0.158	57.10(51.40, 63.40)	54.60(49.65, 61.80)	0.399
LYMPH%	32.90(27.50, 38.20)	36.00(30.65, 38.80)	0.034	32.80(27.60, 37.98)	36.00(31.70, 40.20)	0.100	32.90(27.40, 38.40)	36.05(28.78, 38.98)	0.182
MON%	7.50(6.50, 8.60)	7.30(6.10, 8.05)	0.134	7.80(6.80, 8.90)	7.50(6.40, 8.20)	0.308	7.00(5.90, 7.90)	6.55(5.88, 8.00)	0.764
EOS%	2.20(1.40, 3.30)	1.70(1.25, 2.50)	0.050	2.40(1.60, 3.60)	2.00(1.40, 3.75)	0.581	1.80(1.30, 2.70)	1.65(1.10, 2.13)	0.127
BAS%	0.50(0.40, 0.70)	0.50(0.40, 0.70)	0.788	0.60(0.40, 0.70)	0.50(0.40, 0.80)	0.807	0.40(0.30, 0.60)	0.50(0.33, 0.68)	0.183
NEU#(× 10^*^9/L)	3.75(3.00, 4.82)	3.86(3.09, 4.52)	0.987	3.65(3.00, 4.70)	3.56(3.09, 4.26)	0.953	4.09(3.06, 5.24)	3.94(2.97, 4.66)	0.808
LYMPH#(× 10^a^9/L)	2.16(1.74, 2.65)	2.64(2.04, 3.00)	0.002	2.15(1.71, 2.60)	2.69(1.97, 3.03)	0.020	2.19(1.83, 2.74)	2.58(2.06, 2.99)	0.078
MON#(× 10^*^9/L)	0.50(0.42, 0.60)	0.53(0.42, 0.62)	0.692	0.52(0.43, 0.62)	0.57(0.41, 0.66)	0.621	0.47(0.39, 0.57)	0.51(0.43, 0.58)	0.464
EOS#(× 10^*^9/L)	0.15(0.10, 0.23)	0.14(0.08, 0.18)	0.174	0.16(0.10, 0.24)	0.15(0.10, 0.27)	0.909	0.13(0.08, 0.20)	0.11(0.06, 0.16)	0.219
BAS#(× 10*9/L)	0.04(0.02, 0.05)	0.04(0.03, 0.05)	0.293	0.04(0.03, 0.05)	0.04(0.03, 0.05)	0.629	0.03(0.02, 0.04)	0.04(0.02, 0.05)	0.105

Euthyroid indicates that FT3, FT4 and TSH levels are in the normal range.Hypothyroidism included newly diagnosed overt hypothyroidism and newly diagnosed subclinical hypothyroidism. *BMI* body mass index, *NC* neck circumference, *WC* waist circumference, *ESS* Epworth sleepiness scale, *AHI* apnea hypopnea index, *ODI* oxygen desaturation index, *ArI* arousal index, *meanSpO2* mean percutaneous oxygen saturation, *minSpO2* minimum percutaneous oxygen saturation, *T90* proportion of cumulative sleep time with SpO2 below 90% in total sleep time, *FT3* free triiodotironine, *FT4* free thyroxine, *TSH* thyroid stimulating hormone, *WBC#* white blood cell count, *RBC#* red blood cell count, *HGB* hemoglobin level, *PLT#* platelet count, *NEU%* neutrophil percentage, *LYMPH%* lymphocyte percentage, *MON%* monocyte percentage, *EOS%* eosinophil cell percentage, *BAS%* basophil cell percentage, *NEU#* neutrophil count, *LYMPH#* lymphocyte count, *MON#* monocyte count, *EOS#* eosinophil cell count, *BAS#* basophil cell count

#### Gender subgroup

The 920 OSA patients included 617 male and 303 female subjects (Table 1). In males, AHI, ODI, and T90 were higher in those with hypothyroidism than in patients with normal thyroid function, although the difference was not statistically significant. Moreover, meanSpO_2_ and miniSpO_2_ were slightly lower in the hypothyroid group than in the control group. Furthermore, we found that the lymphocyte count was significantly higher in OSA patients with hypothyroidism than in controls (*p* = 0.020). The lymphocyte count of the female OSA population was higher in patients with hypothyroidism than in controls, even though the difference was nonsignificant (Fig. 2B). In addition, in both male and female OSA patients, a significantly higher ESS score was found in the hypothyroid group than in the euthyroid group.

#### Age subgroup

As shown in Table 2, the included population was predominantly younger patients, and among patients aged < 60 years, the hypothyroid group had a higher BMI. At the same time, in patients younger than 60 years old, the ESS score was significantly higher in the hypothyroidism group than in the euthyroid group (*p* = 0.028). Moreover, the hypothyroidism group tended to have a higher lymphocyte level than the control group (*p* = 0.011) (Fig. 2C).

**Table 2:** Characteristics of the clinical data of the hypothyroidism group and the euthyroid group of patients with OSA, using 60 years of age as a cutoff

	Age＜60(n=771)			Age≥60(*n*=149)		
	Euthyroid	Hypothyroidism	P	Euthyroid	Hypothyroidism	P
Participant	736.00(95.46%)	35.00(4.54%)	-	143.00(95.97%)	6.00(4.03%)	-
Age	39.26±10.32	37.83±11.22	0.426	66.83±5.38	63.83±2.40	0.177
BMI	33.09±8.05	36.00±9.15	0.038	28.71±4.86	27.67±2.47	0.606
NC	42.17±5.26	41.89±4.19	0.756	40.34±3.89	39.83±2.99	0.752
WC	108.96±16.83	113.24±18.16	0.143	103.86±12.89	99.00±6.23	0.361
ESS	6.00(3.00，12.00)	10.00(4.00，14.00)	0.028	8.00(4.00，13.00)	11.00(9.25，15.50)	0.143
AHI	46.40(21.63，72.18)	39.60(12.50，78.70)	0.682	42.10(19.90，65.20)	47.00(30.63，62.45)	0.674
ODI	38.25(17.20，68.53)	36.00(14.50，68.40)	0.875	36.50(14.30，54.90)	41.60(28.53，57.33)	0.395
ArI	24.20(13.40，45.48)	23.90(12.80，50.90)	0.775	20.60(12.00，35.50)	27.15(11.83，39.78)	0.746
meanSpO2	94.00(92.00，96.00)	94.00(90.00，95.00)	0.418	94.00(92.00，95.00)	93.00(91.75，95.00)	0.822
miniSpO2	78.00(65.00，85.00)	76.00(60.00，86.00)	0.366	78.00(71.00，85.00)	69.00(61.00，75.00)	0.051
T90	4.70(0.30，24.78)	3.90(0.30，35.90)	0.841	6.50(0.60，21.20)	16.40(3.48，26.93)	0.281
FT3	4.54(4.21，4.95)	4.36(4.05，4.90)	0.158	4.23(3.88，4.61)	4.46(4.20，4.76)	0.257
FT4	12.40(11.54，13.37)	11.91(10.90，13.45)	0.096	12.18(11.38，13.45)	11.90(10.29，13.49)	0.481
TSH	1.88(1.31，2.58)	5.78(5.21，7.39)	<0.001	1.79(1.30，2.38)	6.60(5.63，10.14)	<0.001
WBC#(×10*9/L)	6.84(5.72，8.14)	7.51(6.02，8.88)	0.131	6.51(5.23，8.02)	6.56(5.61，7.81)	0.862
RBC#(×10*12/L)	4.95(4.61，5.29)	4.86(4.64，5.26)	0.448	4.49(4.17，4.83)	4.45(4.18，5.06)	0.798
HGB(g/L)	148.00(135.25，158.00)	144.00(126.00，155.00)	0.065	138.00(128.00，148.00)	138.50(127.25，156.25)	0.615
PLT#(×10*9/L)	250.00(216.25，293.00)	253.00(215.00，297.00)	0.437	215.00(170.00，253.00)	235.00(188.00，261.00)	0.579
NEU%	55.60(50.30，61.30)	54.50(49.30，60.90)	0.446	59.60(53.60，65.90)	53.85(47.10，59.85)	0.087
LYMPH%	33.45(28.53，38.60)	36.00(31.40，39.20)	0.128	29.20(22.70，35.90)	36.10(29.53，41.35)	0.061
MON%	7.40(6.40，8.60)	7.20(6.00，8.00)	0.072	7.90(6.70，8.90)	8.10(6.70，9.73)	0.657
EOS%	2.20(1.50，3.40)	1.80(1.30，2.60)	0.079	2.00(1.20，3.00)	1.40(1.00，2.43)	0.359
BAS%	0.50(0.40，0.70)	0.50(0.40，0.70)	0.973	0.50(0.30，0.60)	0.55(0.30，0.88)	0.477
NEU#(×10*9/L)	3.73(3.00，4.79)	3.86(3.03，4.55)	0.745	3.87(3.00，4.95)	3.55(2.94，4.15)	0.451
LYMPH#(×10*9/L)	2.22(1.83，2.67)	2.64(2.03，3.00)	0.011	1.83(1.34，2.35)	2.51(1.91，2.93)	0.055
MON#(×10*9/L)	0.50(0.42，0.60)	0.53(0.42，0.60)	0.890	0.49(0.41，0.60)	0.56(0.44，0.64)	0.374
EOS#(×10*9/L)	0.15(0.10，0.24)	0.15(0.09，0.18)	0.242	0.13(0.07，0.19)	0.09(0.06，0.16)	0.351
BAS#(×10*9/L)	0.04(0.03，0.05)	0.04(0.03，0.05)	0.496	0.03(0.02，0.04)	0.04(0.02，0.05)	0.330

Euthyroid indicates that FT3, FT4 and TSH levels are in the normal range. Hypothyroidism included newly diagnosed overt hypothyroidism and newly diagnosed subclinical hypothyroidism. *BMI* body mass index, *NC* neck circumference, *WC* waist circumference, *ESS* Epworth sleepiness scale, *AHI* apnea hypopnea index, *ODI* oxygen desaturation index, *Arl* arousal index, *meanSpO2* mean percutaneous oxygen saturation, *minSpo2* minimum percutaneous oxygen saturation, *T90* proportion of cumulative sleep time with SpO2 below 90% in total sleep time, *FT3* free triiodotironine, *FT4* free thyroxine, *TSH* thyroid stimulating hormone, *WBC#* white blood cell count, *RBC#* red blood cell, *HGB* hemoglobin level, *PLT#* platelet count, *NEU%* neuthrophil percentage, *LYMPH%* lymphocyte percentage, *MON%* monocyte percentage, *EOS%* eosinophil cell percentage, *BAS%* basophil cell percentage, *NEU#* neutrophil count, *LYMPH#* lymphocyte count, *MON#* monocyte count, *EOS#* eosinophil cell count, *BAS#* basophil cell count

#### AHI subgroup

According to the AHI grouping (Table 3), we found that in patients with mild-moderate OSA, lymphocyte counts were higher in hypothyroid patients than in controls, and the differences were statistically significant (Fig. 2D). In patients with severe OSA, the absolute value of lymphocytes was higher in the hypothyroid group than in the euthyroid group, but the difference was not statistically significant (Fig. 2D).

**Table 3:** Characteristics of the clinical data of the hypothyroidism group and the euthyroid group of patients with OSA, using AHI as a cutoff

	AHI ≤ 30 (*n* = 328)			AHI > 30 (*n* = 592)		
	Euthyroid	Hypothyroidism	P	Euthyroid	Hypothyroidism	P
Participant	313.00(95.43%)	15.00(4.57%)	-	566.00(95.61%)	26.00(4.39%)	-
Age	42.17 ± 14.80	34.27 ± 12.61	0.043	44.61 ± 13.57	45.88 ± 13.05	0.640
BMI	31.22 ± 7.66	35.48 ± 6.48	0.035	33.02 ± 7.79	34.38 ± 10.26	0.508
NC	40.10 ± 3.67	40.60 ± 3.18	0.602	42.85 ± 5.51	42.15 ± 4.47	0.526
WC	103.81 ± 16.02	109.23 ± 14.80	0.200	110.52 ± 16.06	112.27 ± 19.27	0.591
ESS	5.00(2.00, 8.00)	6.00(3.00, 10.00)	0.496	8.00(4.00, 14.00)	12.00(10.00, 15.25)	0.006
AHI	16.90(10.80, 23.25)	12.30(8.70, 17.40)	0.100	65.75(47.05, 79.28)	64.20(40.88, 85.15)	0.669
ODI	12.40(7.60, 18.35)	14.20(8.20, 19.00)	0.751	58.65(39.83, 75.65)	54.55(35.93, 78.30)	0.965
ArI	12.10(8.10, 17.75)	12.80(7.90, 16.00)	0.616	36.40(21.78, 54.15)	36.40(23.73, 61.00)	0.766
meanSpO2	96.00(95.00, 96.00)	95.00(94.00, 96.00)	0.275	93.00(90.00, 94.00)	92.00(89.75, 94.25)	0.617
miniSpO2	85.00(82.00, 89.00)	86.00(80.00, 87.00)	0.272	71.00(59.75, 79.00)	66.00(52.25, 73.75)	0.069
T90	0.10(0.00, 1.10)	0.40(0.00, 1.50)	0.500	14.65(4.68, 37.73)	19.50(4.35, 36.85)	0.747
FT3	4.44(4.11, 4.82)	4.48(4.06, 4.99)	0.970	4.54(4.17, 4.95)	4.39(4.02, 4.76)	0.256
FT4	12.25(11.41, 13.38)	12.02(11.31, 13.46)	0.706	12.42(11.55, 13.39)	11.64(10.80, 13.12)	0.050
TSH	1.90(1.34, 2.59)	5.36(5.21, 5.97)	< 0.001	1.85(1.30, 2.52)	6.31(5.35, 7.97)	< 0.001
WBC#(× 10*9/L)	6.62(5.38, 8.20)	7.72(6.29, 9.41)	0.054	6.85(5.76, 8.05)	7.34(5.89, 8.11)	0.734
RBC#(× 10*12/L)	4.70(4.42, 5.07)	4.79(4.52, 4.94)	0.883	4.97(4.63, 5.30)	4.91(4.53, 5.31)	0.629
HGB(g/L)	140.00(130.00, 151.00)	134.00(122.00, 150.00)	0.407	149.00(137.00, 158.00)	146.00(127.50, 155.50)	0.209
PLT#(× 10*9/L)	245.00(212.00, 293.00)	296.00(253.00, 319.00)	0.019	245.00(210.00, 283.25)	243.00(208.25, 262.00)	0.517
NEU%	55.90(50.85, 61.90)	54.00(48.10, 62.30)	0.391	56.25(50.68, 62.50)	55.00(49.50, 59.75)	0.272
LYMPH%	33.80(28.65, 37.60)	36.50(28.10, 42.00)	0.197	32.55(27.20, 38.33)	35.25(31.70, 37.88)	0.087
MON%	7.40(6.20, 8.60)	7.20(6.40, 8.40)	0.746	7.60(6.60, 8.70)	7.35(5.75, 7.93)	0.039
EOS%	2.00(1.30, 3.25)	1.60(1.20, 2.20)	0.157	2.20(1.50, 3.30)	1.85(1.30, 2.70)	0.180
BAS%	0.50(0.40, 0.60)	0.50(0.30, 0.60)	0.761	0.50(0.40, 0.70)	0.55(0.48, 0.73)	0.568
NEU#(× 10*9/L)	3.74(2.90, 4.77)	3.86(3.42, 4.66)	0.380	3.78(3.05, 4.84)	3.89(2.75, 4.37)	0.501
LYMPH#(× 10*9/L)	2.13(1.76, 2.64)	2.64(2.31, 3.00)	0.007	2.19(1.73, 2.65)	2.47(2.00, 3.01)	0.075
MON#(× 10*9/L)	0.49(0.40, 0.59)	0.58(0.50, 0.67)	0.014	0.51(0.43, 0.61)	0.49(0.39, 0.59)	0.186
EOS#(× 10*9/L)	0.14(0.09, 0.22)	0.12(0.09, 0.16)	0.569	0.15(0.10, 0.24)	0.15(0.07, 0.19)	0.236
BAS#(× 10*9/L)	0.03(0.02, 0.05)	0.03(0.02, 0.05)	0.620	0.04(0.03, 0.05)	0.04(0.03, 0.05)	0.332

Euthyroid indicates that FT3, FT4 and TSH levels are in the normal range. Hypothyroidism included newly diagnosed overt hypothyroidism and newly diagnosed subclinical hypothyroidism. *BMI* body mass index, *NC* neck circumference, *WC* waist circumference, *ESS* Epworth sleepiness scale, *AHI* apnea hypopnea index, *ODI* oxygen desaturation index, *ArI* arousal index, *meanSpO2* mean percutaneous oxygen saturation, *minSpO2* minimum percutaneous oxygen saturation, *T90* proportion of cumulative sleep time with SpO2 below 90% in total sleep time, *FT3* free triiodotironine, *FT4* free thyroxine, *TSH* thyroid stimulating hormone, *WBC#* white blood cell count, *RBC#* red blood cell count, *HGB* hemoglobin level, *PLT#* platelet count, *NEU%* neutrophil percentage, *LYMPH%* lymphocyte percentage, *MON%* monocyte percentage, *EOS%* eosinophil cell percentage, *BAS%* basophil cell percentage, *NEU#* neutrophil count, *LYMPH#* lymphocyte count, *MON#* monocyte count, *EOS#* eosinophil cell count, *BAS#* basophil cell count

### Multivariate analysis of independent predictors of hypothyroidism

Based on the results of the above data analysis and clinical significance, these indicators of sex, age, AHI, BMI, ESS, and Lymph# were selected for multifactorial logistic analysis to explore the relevant influencing factors of hypothyroidism. Binary logistic regression analysis showed that sex, ESS, and lymphocyte count were independent predictors of OSA combined with hypothyroidism (*p* = 0.030, 0.008, and 0.005, respectively) (Table 4). The prevalence of hypothyroidism increased by 67.3% if the lymphocyte count increased by 1.0 × 10*9/L.

**Table 4:** Multivariate analysis to determine the independent predictors of hypothyroidism in OSA patients

	Odds Ratio	95% Confidence Interval	P
Sex	0.450	0.219–0.925	0.030
Age	1.000	0.974–1.027	0.982
BMI	1.020	0.974–1.067	0.398
ESS	1.071	1.018–1.126	0.008
AHI	0.998	0.987–1.009	0.721
LYMPH#(10*9/L)	1.673	1.168–2.396	0.005

*BMI* body mass index, *ESS* Epworth sleepiness scale, *AHI* apnea hypopnea index, *LYMPH#* lymphocyte count

### ROC analysis for lymphocyte count to predict hypothyroidism in OSA patients

**Fig. 3:**
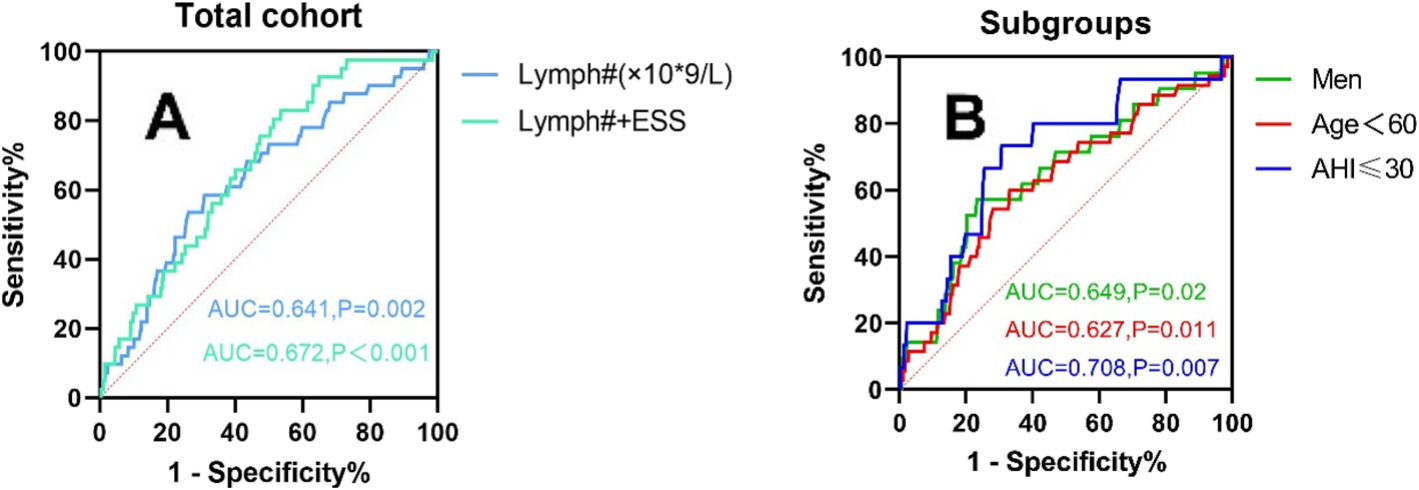
**A** Receiver operating characteristic (ROC) curve analysis for lymphocyte count and the combination indicator to predict hypothyroidism. **B** The discriminatory capacity of lymphocyte count for distinguishing between patients with and without hypothyroidism in different subgroups of OSA patients

**Fig. 4 A:**
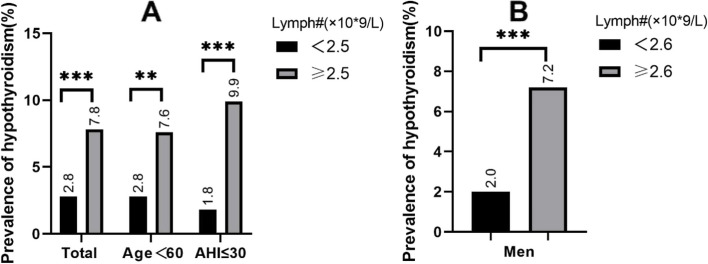
Prevalence of hypothyroidism in different OSA groups when a lymphocyte count ≥ 2.5 (× 10*9/L) was used as the cutoff value. (*p < 0.05,**p < 0.01,***p < 0.001). **B** Prevalence of hypothyroidism in male OSA patients when a lymphocyte count ≥ 2.6 (× 10*9/L) was used as the cutoff value.(*p < 0.05,**p < 0.01,***p < 0.001)

ROC curve analysis was performed to determine the cutoff lymphocyte count to predict the presence of hypothyroidism in OSA patients (Fig. 3). In the present study, lymphocytes showed a significantly positive correlation with hypothyroidism in OSA patients. Its optimal cutoff point for diagnosing hypothyroidism in the total OSA population was 2.5 (× 10*9/L). There was a significant difference in the prevalence of hypothyroidism between the two groups of OSA patients when using 2.5 (× 10*9/L) as the cutoff value. ROC analysis showed no significant increase in the diagnostic accuracy of the combined index of lymphocyte count and ESS compared to the single index of lymphocytes. Significant differences in the prevalence of hypothyroidism were shown in different OSA populations when using the lymphocyte optimal cutoff point as a dividing line (Fig. 4).

### Association between lymphocyte counts and PSG parameters

In male patients(*n* = 617), grouped according to lymphocyte levels, there was a significant difference between the two groups regarding AHI and ODI (*p* = 0.038,0.017, respectively). Compared to controls, male OSA patients were more likely to experience more sleep apnea and oxygen reduction events when lymphocyte levels were higher (Table 5).

**Table 5:** Differences in PSG parameters were compared according to lymphocyte groupings in male patients

	LYMPH# (× 10*9/L)		
	< 2.6	≥ 2.6	P
Participant	458.00(74.23%)	159.00(25.77%)	-
AHI	54.10(27.18, 72.55)	60.70(35.40, 79.10)	0.038
ODI	46.05(20.28, 69.10)	55.40(32.90, 77.20)	0.017
ArI	29.20(16.30, 46.85)	34.40(20.40, 50.90)	0.108
meanSpO2	94.00(91.00, 95.00)	93.00(91.00, 95.00)	0.602
miniSpO2	76.00(62.00, 84.00)	73.00(63.00, 82.00)	0.272
T90	9.00(0.79, 30.93)	9.10(2.20, 33.30)	0.255

*AHI* apnea hypopnea index, *ODI* oxygen desaturation index, *ArI* arousal index, *meanSpO2* mean percutaneous oxygen saturation, *minSpO2* minimum percutaneous oxygen saturation, *T90* proportion of cumulative sleep time with SpO2 below 90% in total sleep time

## Discussion

Patients with OSA and hypothyroidism share common clinical manifestations, such as excessive daytime sleepiness, apathy, decreased cognitive function, obesity, and decreased libido [15]. Numerous studies have shown a strong correlation between OSA and hypothyroidism. For example, Jha A et al. reported a 30% prevalence of OSA in newly diagnosed hypothyroidism [16]. Sorensen et al. claimed the prevalence of OSA to be 25%-50% in patients with overt hypothyroidism [17]. In a meta-analysis, Zhang M et al. concluded that the prevalence of clinical hypothyroidism in OSA patients was 8.12 ± 7.13%, and the prevalence of subclinical hypothyroidism was 11.07 ± 8.49% [18]. This shows that there is a close association between OSA and hypothyroidism.

Studies have suggested that hypothyroidism can lead to OSA through the following mechanisms: 1) infiltration of soft tissues by mucopolysaccharides aggravates airway narrowing [19]; 2) muscle dysfunction leads to hypotonia of the respiratory muscles [20, 21]; and 3) a low metabolic rate leads to obesity, and further fat accumulation in the neck and abdomen causes obesity hypoventilation [22]. We similarly observed a higher BMI in the hypothyroid group than controls in the total OSA population (34.78 ± 8.99 vs 32.38 ± 7.79, *p* = 0.055), although there was no significant difference.

Studies have shown that intermittent hypoxia also appears to have a negative effect on thyroid function. Elevated plasma IL-6 levels and decreased IL-10 levels support the prevalent activation of Th1-type cytokine patterns in OSA patients, suggesting the presence of Th1 cell activation in OSA patients [23]. Cytokines secreted by Th1 cells are also common in patients with Hashimoto's thyroiditis [24], and peripheral Th1 cells are more abundant in patients with severe Hashimoto's thyroiditis [25]. In Hashimoto's thyroiditis, Th1 cells can mediate an autoimmune response in the thyroid, with intense inflammatory infiltration and further thyroid destruction [26].

In addition to Th1 cells, several studies have shown that Th17 is closely associated with OSA and hypothyroidism. The findings showed that the proportion of peripheral blood Th17 cells and the relative expression of RORγt mRNA (RORγt is a crucial nuclear transfer factor for Th17 cell differentiation and secretion of IL-17A, which induces differentiation and maturation of Th0 cells into Th17 cells.) were higher in OSA patients than in controls and correlated with the severity of OSA [27]. Similarly, Ye et al. found higher levels of Th17 cells in the peripheral blood of patients with severe OSA compared to the mild group and controls. This result suggests that increased Th17 cell differentiation correlates with OSA severity [28]. Serum IL-17A levels were elevated in OSA patients and positively correlated with AHI [29]. Furthermore, cytology experiments revealed that a hypoxic environment promotes Th17 cell differentiation [30]. Related studies suggest that intermittent hypoxia in OSA may promote increased differentiation of Th17 cells through the hypoxia-inducible factor 1 (HIF-1) and NF-κB pathways [27]. Similarly, in patients with HT, the number of Th17 lymphocytes and Th17 cytokines in the peripheral blood and thyroid were increased [31]. Horie et al. found increased Th17 cells in the thyroid gland in a mouse model of Hashimoto's thyroiditis, confirming the importance of Th17 cells in autoimmune thyroiditis [32].

Therefore, we speculated that lymphocytes might be involved in the process of hypothyroidism in patients with OSA. These findings suggest that intermittent hypoxia may induce lymphocyte activation and thus promote thyroid immune responses, which may contribute to hypothyroidism. Thus, intermittent hypoxia may be an important initiating factor in the development of hypothyroidism, but its intrinsic connection still needs to be further explored. The above findings imply that there may be a bidirectional effect between OSA and hypothyroidism, rather than just hypothyroidism, as we know it is able to cause OSA.

In the present study, we observed a prevalence of hypothyroidism of 4.46% in the OSA population. The prevalence in the male and female patient groups was 3.4% and 6.6%, respectively. Because subclinical hypothyroidism and overt clinical hypothyroidism are different stages of the same disease, the process from subclinical hypothyroidism to clinical hypothyroidism is a continuous progression of the disease [33]. It has been reported that approximately 2%-5% of patients with subclinical hypothyroidism may progress to overt hypothyroidism each year [33]. Therefore, in this study, we categorized subclinical hypothyroidism and hypothyroidism in the same group. We further found that lymphocyte levels were apparently higher in the hypothyroid group than in the euthyroid group, although the difference was insignificant in female OSA patients. Except for lymphocytes, ESS scores were significantly higher in patients with hypothyroidism than in the euthyroid group in the OSA population. Lymphocyte level and ESS score can be used as independent predictors of hypothyroidism in patients with OSA. In the subgroup analysis performed, it was found that the number of lymphocytes was distinctly higher in the hypothyroid group than in the control group in the OSA population younger than 60 years old and patients with mild to moderate OSA, and the difference was statistically significant. Based on the above subgroups, the optimal threshold for lymphocyte count for the diagnosis of hypothyroidism fluctuated between 2.5–2.6 (× 10*9/L) in the OSA population. These results indicate that lymphocyte count can be a valuable biomarker for identifying the presence of hypothyroidism in OSA patients. The prevalence of hypothyroidism in patients with OSA increases as the number of lymphocytes increases. Therefore, lymphocyte levels can be used to estimate a patient's risk of developing hypothyroidism in the future. However, further research is needed to validate these findings and explore the underlying mechanisms of this association.

It is well known that hypothyroidism is one of the risk factors for OSA. Several studies have shown that LT4 therapy can reduce or even eliminate nocturnal apnea for patients with sleep apnea in combination with hypothyroidism [17]. Levothyroxine replacement therapy in patients with OSA combined with subclinical hypothyroidism may reduce the tendency to sleep [34]. These results support the perspective that thyroid function should be investigated in all OSA patients, at least in those with a high risk of hypothyroidism.

There are several limitations to our study. First, some OSA patients were excluded from the study due to incomplete data, resulting in a reduced sample size. Second, because this study was a cross-sectional design, long-term follow-up of the subjects was unrealistic, so their stable thyroid function status could not be known. Third, we did not evaluate the effect of CPAP or hormone supplementation therapy on lymphocyte levels in patients, which requires a prospective interventional study. Fourth, Serum concentrations of serum thyroid peroxidase (TPO) and thyroglobulin antibodies were not further determined to assess the prevalence of autoimmune thyroid disease. Last but not least, this study failed to further analyze the role played by lymphocyte subtypes and cytokines in hypothyroidism in patients with OSA.

## Conclusion

In brief, the present study aimed to assess the correlation between lymphocyte count and hypothyroidism in patients with OSA. Based on our findings, lymphocyte levels tend to be higher in patients with OSA who also have hypothyroidism, and this association is particularly significant in the overall OSA population and male patients. Furthermore, we discovered that lymphocyte count independently predicts the development of hypothyroidism in patients with OSA, which has not been previously reported. Therefore, a simple blood test for lymphocyte count could indicate the occurrence of hypothyroidism in OSA patients, proving valuable in clinical practice.

## Data Availability

No datasets were generated or analysed during the current study.
